# *CAMK2N1/RUNX3* methylation is an independent prognostic biomarker for progression-free and overall survival of platinum-sensitive epithelial ovarian cancer patients

**DOI:** 10.1186/s13148-021-01006-8

**Published:** 2021-01-22

**Authors:** Karolin Heinze, Matthias Rengsberger, Mieczyslaw Gajda, Lars Jansen, Linea Osmers, Leticia Oliveira-Ferrer, Barbara Schmalfeldt, Matthias Dürst, Norman Häfner, Ingo B. Runnebaum

**Affiliations:** 1 Department of Gynecology and Reproduction Medicine, Jena University Hospital–Friedrich Schiller University Jena, 07747 Jena, Germany; 2 Department of Forensic Medicine, Section of Pathology, Jena University Hospital – Friedrich Schiller University Jena, 07747 Jena, Germany; 3 Department of Gynecology, University Medical Center Hamburg-Eppendorf, 20246 Hamburg, Germany

**Keywords:** Epithelial ovarian cancer, Prognostic marker, DNA methylation, RUNX3, CAMK2N1 (CaMKIINα)

## Abstract

**Background:**

To date, no predictive or prognostic molecular biomarkers except *BRCA* mutations are clinically established for epithelial ovarian cancer (EOC) despite being the deadliest gynecological malignancy. Aim of this biomarker study was the analysis of DNA methylation biomarkers for their prognostic value independent from clinical variables in a heterogeneous cohort of 203 EOC patients from two university medical centers.

**Results:**

The marker combination *CAMK2N1/RUNX3* exhibited a significant prognostic value for progression-free (PFS) and overall survival (OS) of sporadic platinum-sensitive EOC (*n* = 188) both in univariate Kaplan–Meier (LogRank *p* < 0.05) and multivariate Cox regression analysis (*p* < 0.05; hazard ratio HR = 1.587). *KRT86* methylation showed a prognostic value only in univariate analysis because of an association with FIGO staging (Fisher’s exact test *p* < 0.01). Thus, it may represent a marker for EOC staging. Dichotomous prognostic values were observed for *KATNAL2* methylation depending on *BRCA* aberrations. *KATNAL2* methylation exhibited a negative prognostic value for PFS in sporadic EOC patients without *BRCA1* methylation (HR 1.591, *p* = 0.012) but positive prognostic value in sporadic EOC with *BRCA1* methylation (HR 0.332, *p* = 0.04) or *BRCA*-mutated EOC (HR 0.620, n.s.).

**Conclusion:**

The retrospective analysis of 188 sporadic platinum-sensitive EOC proved an independent prognostic value of the methylation marker combination CAMK2N1/RUNX3 for PFS and OS. If validated prospectively this combination may identify EOC patients with worse prognosis after standard therapy potentially benefiting from intensive follow-up, maintenance therapies or inclusion in therapeutic studies. The dichotomous prognostic value of *KATNAL2* should be validated in larger sample sets of EOC.

## Background

Despite all medical efforts and advances, epithelial ovarian cancer (EOC) remains the most lethal gynecological malignancy. In Germany, this disease accounts for 3.2% of all newly diagnosed cancer cases and 5.3% of all cancer-related death of women [1]. The lifetime risk of developing this cancer entity lies by 1–2%. There are several factors increasing or decreasing the lifetime risk. Demographic, environmental and hormonal facts possibly add to the risk. Additionally, various familiar factors influence the chances of developing EOC. Family cancer syndromes predispose patients for the development of this malignancy. A number of syndromes are associated with ovarian cancer, the hereditary breast and ovarian cancer (HBOC) syndrome being the most prominent one [2]. Within this syndrome, the genes *BRCA1* and *BRCA2* are frequently mutated [3, 4]. Germline mutations account for 15–25% of all ovarian carcinoma [5, 6] while somatic mutations of the *BRCA* genes are additionally detected in 5–6% of EOC [7, 8]. The five-year survival rate of EOC lies between 35 and 45% [1, 9–11]. These low survival numbers can be explained by the lack of early detection methods resulting in the frequent diagnosis of progressed tumor stages (≥ FIGO III). Standard therapy comprises surgery to reduce the tumor burden combined with platin-taxane-based chemotherapy. A successful surgical intervention is crucial for improved survival. A macroscopic completely resected tumor is most preferable and improves the odds of survival and therapy success [12, 13]. Whereas approximately 20% of patients exhibit intrinsic resistant EOC most tumors of initially sensitive patients develop platin resistance and relapse within a median of 18 month [14, 15]. Neither prognostic markers for EOC nor predictive markers for standard chemotherapy are clinically established. Patients with *BRCA* mutations tend to respond better to platin and show improved progression-free survival but this does not necessarily translate into improved long-term overall survival [16, 17]. Additionally, *BRCA* mutations or homologous repair defects (HRD) can identify patients benefitting from PARPi treatment [18–21]. However, even patients with HRD-proficient tumors can be successfully treated with PARPi [19, 22] pointing to the potential requirement to establish better predictive marker. Moreover, no predictive marker is defined for anti-angiogenesis treatment with Bevacizumab. Thus, in the absence of predictive biomarker for any adjuvant treatment, prognostic biomarker may help to identify patients with worse outcome benefitting from maintenance treatment with these new treatment options. Both clinical parameters and different molecules or cell populations are targets of intense research to identify and validate prognostic marker. Clinical parameters can be analyzed posttreatment only (resection status, tumor stage, chemotherapeutic response) but are well established [23]. Biomarker may be analyzed pre-therapeutically (blood-based liquid biopsy) or at least before start of adjuvant chemotherapy (tissue-based biomarker) but are far from being implemented in clinical routine. Biomarker classes for liquid biopsies can be circulating nucleic acids, immune parameters, serum proteins or extracellular vesicle carrying miRNA and other molecules [24–28]. Tissue-based marker may consist of aberrant DNA, coding- or non-coding RNA expression or the analysis of intratumoral immune cell populations [7, 29–34]. One DNA aberration which can be used as molecular biomarker is CpG hypermethylation belonging to the field of epigenetics.

Epigenetic changes influence gene expression in a reversible manner without changing the DNA sequence. Histone modification, DNA methylation and, micro-RNA associated silencing are modes of epigenetic alteration in both normal developmental processes and disease, e.g., cancer development [35, 36]. DNA methylation is a stable epigenetic modification catalyzed by DNA-Methyltransferases (DNMT’s) and recognized by methyl-CpG binding proteins (MBDs) resulting in changed chromatin states. Since 5-methyl-cytosines and the DNA itself are highly stable, efforts to use these as biomarker are the focus of current medical research [37–39]. Epigenetic changes are seen in various tumor entities, ovarian cancer being one of them [40]. Currently, aberrant DNA methylation in EOC is detected using different approaches such as target-specific methylation-specific PCR (MSP) [41] or whole genome approaches [42–44]. A large number of genes are affected by DNA methylation in EOC [40]. For instance, tumor suppressor genes such as *BRCA1*, *RASSF1A* [45] or *MLH1* [41, 46]; cell adhesion genes as *ICAM-1* [47] and *CDH1* [48] and DNA repair genes *PALB2* [49] are affected by DNA methylation. *BRCA1* promoter methylation is reported in 10–15% of sporadic OvCa [7, 50–53]. It appears that a reversal of this phenotype can take place over time leading to changed methylation level in relapsed disease [54]. *MLH1* methylation is potentially linked to chemotherapy response or resistance development [46]. However, lack of candidate biomarker validation and low sensitivity or specificity prevents the clinical use of methylation-based biomarker [55].

In previous studies, we screened twelve primary high-grade serous ovarian carcinomas (HGSOC) using genome wide CpG microarrays for differentially methylated genes to identify potential prognostic biomarkers. 37 hypo-/hypermethylated regions were selected to verify promising candidates in a comparative cohort analysis of 36 samples (PFS < 3 years vs. > 3 years). The methylation marker genes *ATL1, CAMK2N1, KATNAL2, KRT86* and *RUNX3* were detected as most promising due to good discriminatory power and the combination *CAMK2N1* and *RUNX3* could identify EOC patients with significantly shortened PFS in univariate analysis [44]. However, the small number of patients and missing multivariate analyzes for the prognostic value related to PFS and OS were limitations of this study. Nevertheless, subsequent functional analyses identified tumor suppressive functions for *CAMK2N1* and a dichotomous role for *RUNX3* transcript variants regulated by DNA methylation [56]. Robust validation and proof of an independent prognostic value are crucial before biomarkers are tested prospectively in a clinical setting. Therefore, this study aimed to validate the previously identified and most promising epigenetic candidate genes—*ATL1*, *CAMK2N1, KATNAL2, KRT86* and *RUNX3*—using a larger patient cohort from two different University hospitals enabling multivariate analyses. To provide further information for patients with a hereditary form of ovarian cancer, a small number of such patients were also included and the *BRCA1* methylation status was additionally evaluated in all samples.

## Results

### Methylation analysis of biomarker candidate genes

Patients from two university hospital centers were included in this retrospective analysis. Inclusion criteria were histologically confirmed primary epithelial ovarian cancer, available fresh frozen tissue and at least 36 months follow-up data. After screening of the accessible information and material, samples were excluded due to underrepresentation of tumor cells (< 10%) and tissue derived from relapse events. A total of 203 tumor samples existed for the validation of biomarker candidate genes. Samples from patients with known or identified *BRCA1/2* mutations (7.5%; *n* = 15) were evaluated in a separate analysis (see below) to exclude any bias caused by the known prognostic value of *BRCA* mutations [16]. Basic clinical data from the analyzed patients are shown in Table 1.

**Table 1:** Clinical characteristics of analyzed EOC patients

Criteria	Sporadic EOC		Mutated EOC	
	n	%	n	%
**FIGO**				
I	13	6.9	3	20.0
II	6	3.2	0	0.0
III/IV	165	87.8	12	80.0
Missing	4	2.1	0	0.0
**Grading**				
G1	6	3.2	3	20.0
G2	29	15.4	2	13.3
G3	147	78.2	10	66.7
Missing	6	3.2	0	0.0
**pT**				
T1a	6	3.2	1	6.7
T1b	1	0.5	0	0.0
T1c	7	3.7	2	13.3
T2a	8	4.3	0	0.0
T2c	7	3.7	0	0.0
T3a	8	4.3	0	0.0
T3b	20	10.6	2	13.3
T3c	129	68.6	10	66.7
Missing	2	1.1	0	0.0
**pN**				
N0	60	31.9	5	33.3
N1	114	60.6	7	46.7
Nx	14	7.4	3	20.0
**pM**				
M0	137	72.9	12	80.0
M1	35	18.6	3	20.0
Mx	16	8.5	0	0.0
**Histology**				
Serous	156	83.0	13	86.7
Endometrioid	15	8.0	0	0.0
Mucinous	3	1.6	2	13.3
Other	14	7.4	0	0.0
**Resection**				
R0	141	75.0	12	80.0
R1/R2	42	22.3	3	20.0
Missing	5	2.7	0	0.0
**Chemotherapy**				
Carbo/Pac	150	79.8	13	86.7
Carbo	8	4.3	0	0.0
Carbo/Pac + x	11	5.9	0	0.0
Carbo + x	8	4.3	1	6.7
Other	3	1.6	0	0.0
None	7	3.7	1	6.7
Missing	1	0.5	0	0.0
**Bevacizumab**				
Yes	29	15.4	5	33.3
No	157	83.5	10	66.7
Missing	2	1.1	0	0.0
**Complete**	**188**	**100.0**	**15**	**100.0**

The cohort from Jena University Hospital (*n* = 101) consists of 65 new patients but also includes 36 samples from our previous study [44]. To enable a correct validation, all samples used for initial marker identification were excluded. To validate the robustness of employed MSP assays and to exclude major bias by tumor heterogeneity, several experiments were done. First of all, a subset of samples analyzed in Häfner et al. [44] was re-tested. Since several years and different other analyses were performed using the very same tumor block, a deeper section of the tumor was analyzed. Secondly, from some patients, multiple biopsy tissues were available. Both analyses revealed a high consistency of the methylation pattern throughout the samples (95% concordance, Cohen’s Kappa = 0.881) proving the validity and robustness of methylation marker detection (Additional file 1: Fig. S1).

Altogether the cohort reflects the typical EOC patient and mainly comprises of late stage HGSOC patients who were treated by macroscopically complete resection and platin-taxane-based chemotherapy (Table 1). However, the cohort is more diverse than the establishing cohort in regard to tumor stage, histology, surgical resection status and adjuvant chemotherapy. Specifically, a subset of patients received maintenance treatment with Bevacizumab, currently standard of care for advanced stage EOC patients in Germany [57]. Evaluating the clinical parameters of the 188 included sporadic EOC patients, we confirmed the prognostic value of the FIGO staging (FIGO I + II vs. FIGO III + IV) and surgery outcome (tumor residual 0 mm vs. < 10 mm/ > 10 mm) for overall survival (OS) and progression-free survival (PFS) (Table 2; Additional file 1: Fig. S2; LogRank *p* < 0.05). High-grade serous EOC tend to show a shorter PFS than other EOC (*p* = 0.08). The differentiation stage of the tumors (G1/2 vs. G3) did not show a prognostic value for PFS or OS.

**Table 2:** Results of univariate LogRank analyses of clinical and molecular variables

Criteria	Status	n	[%]	PFS [month]			OS [month]		
				Median	95% CI	LogRank	Median	95% CI	LogRank
FIGO	I/II	19	10.4	Not reached		***p < 0.001***	145	46.0–244.0	***p = 0.013***
	III/IV	163	89.6	29	25.5–32.5		62	49.4–74.6	
Resection	R0	139	76.8	38	28.3–47.8	***p < 0.001***	78	71.2–84.8	***p < 0.001***
	R1/2	42	23.2	20	13.9–26.1		49	41.2–56.8	
Histology	Serous	154	82.8	30	26.6–33.4	*p* = 0.075	78	34.9–121.1	*p* = 0.581
	Non-serous	32	17.2	58	0–121.1		64	54.7–73.3	
Methylation	No marker	77	41.4	30	14.8–45.2	*p* = 0.147	78	52.5–103.6	*p* = 0.195
	≥ 1 marker	109	58.6	31	27.4–34.7		62	50.1–73.9	
*CAMK2N1*	U	170	91.4	31	25.2–36.8	***p = 0.021***	74	62.3–85.2	*p* = 0.06
	M	16	8.6	16	9.3–22.8		47	31.2–62.8	
*ATL1*	U	159	85.5	31	25.2–36.8	*p* = 0.179	64	52.9–75.1	*p* = 0.273
	M	27	14.5	27	20.2–33.8		65	26.6–103.4	
*KATNAL2*	U	111	59.7	33	23.3–42.1	*p* = 0.125	78	55.7–100.3	*p* = 0.161
	M	75	40.3	29	24.6–33.5		59	52.6–65.4	
*KRT86*	U	143	76.9	33	25.1–40.9	***p = 0.001***	64	50.1–77.9	*p* = 0.290
	M	43	23.1	26	18.1–33.1		73	54.4–91.6	
*RUNX3*	U	160	86.0	31	26.9–35.1	*p* = 0.126	73	62.3–83.7	*p* = 0.179
	M	26	14.0	21	4.7–37.2		55	33.0–77	
*CAMK2N1/RUNX3*	U	152	81.7	32	26.0–38.0	***p = 0.032***	74	61.7–86.3	***p = 0.045***
	M	34	18.3	21	3.8–38.1		57	43.6–70.4	
*CAMK2N1/KRT86*	U	133	71.5	37	25.9–48.1	***p < 0.001***	74	58.0–90.0	*p* = 0.128
	M	53	28.5	26	18.5–32.8		60	42.9–77.1	
*CAMK2N1/KRT86/RUNX3*	U	123	66.1	37	19.2–54.8	***p = 0.001***	74	57.0–91.0	*p* = 0.106
	M	63	33.9	29	23.0–35.0		60	43.1–76.9	
*BRCA1*	U	166	89.7	31	27.2–34.8	*p* = 0.626	73	61.4–84.6	*p* = 0.308
	M	19	10.3	25	11.8–37.4		57	43.8–70.2	

LogRank results with significance are marked in bold. U, unmethylated; M, methylated. Marker combinations are positive (methylated) if at least one single marker is methylated (<OR> combinations) otherwise the combination is scored as unmethylated

**Fig. 1:**
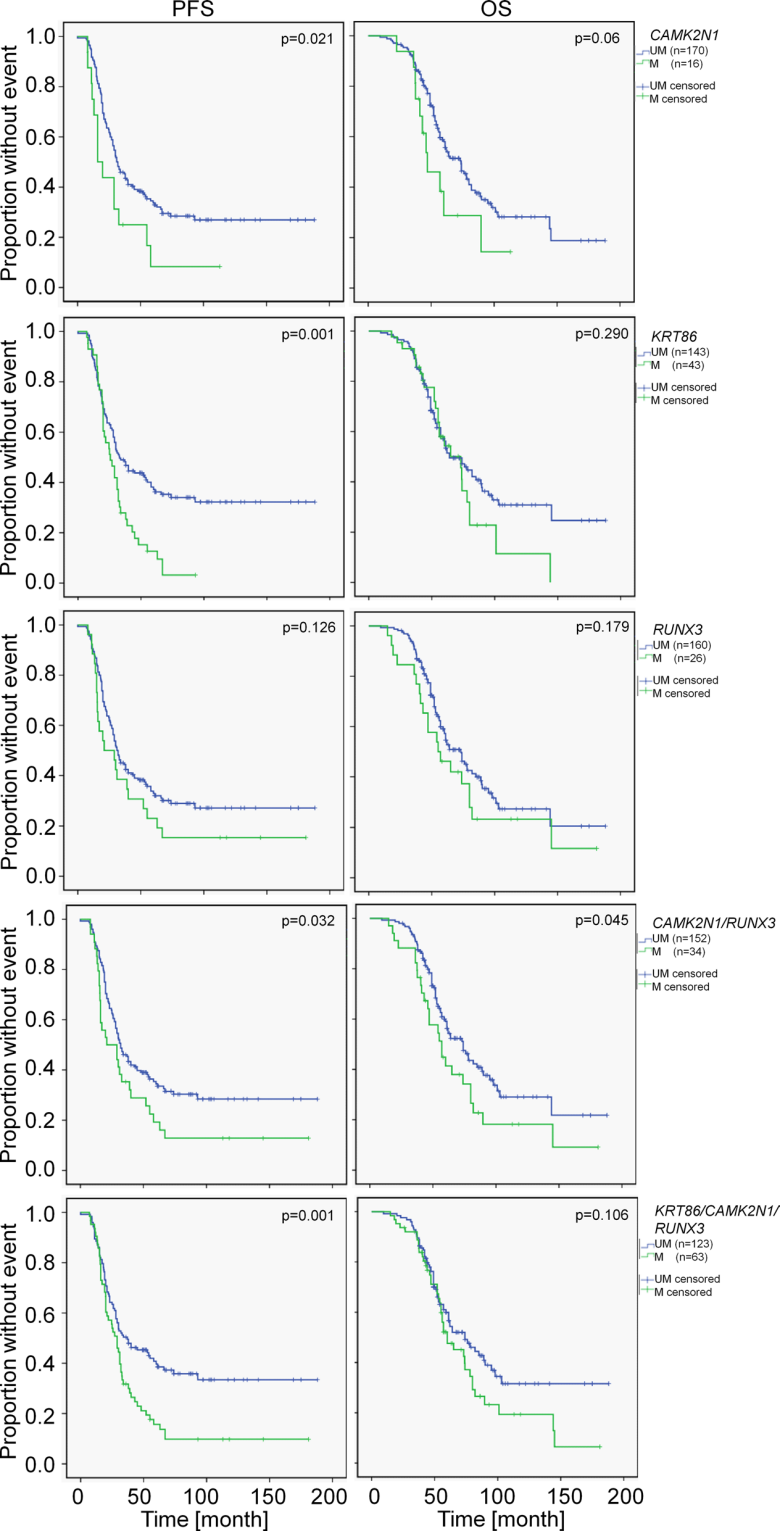
Kaplan–Meier plots for PFS and OS of sporadic EOC patients stratified by methylation markers. LogRank test was utilized for statistical evaluation

Table 2 summarizes, in addition to the clinical parameters, the median and 95% confidence interval of PFS and OS as determined by univariate Kaplan–Meier analyzes for patients stratified by methylation biomarkers or combinations thereof. Combinations reflect the methylation status of at least one included marker (logical <OR> combination). Methylation of CAMK2N1, KRT86 and the combinations CAMK2N1/RUNX3 or CAMK2N1/RUNX3/KRT86 stratified patients into groups with significantly different PFS (Fig. 1, Table 2).

This confirmed our earlier results for these markers, whereas the prognostic value of the singular *RUNX3* methylation could not be validated (Fig. 1, Table 2). Albeit *KRT86* methylation showed the most significant discriminatory power in univariate analysis, it exhibited no independent prognostic value (*p* = 0.055, Table 3). This is mainly related to the exclusive methylation in late stage EOC and the resulting significant association of *KRT86* methylation with FIGO staging (Fisher’s exact test, *p* < 0.01). The present large cohort with overall survival data enabled the additional determination of prognostic effects related to overall survival. Only the combination of methylation markers *CAMK2N1/RUNX3* exhibited a significant prognostic value for OS (Fig. 1, Table 2). The other methylation marker could not significantly differentiate between patient groups with varying OS (Fig. 1, Table 2). However, patients differentiated by *CAMK2N1* methylation as singular marker exhibited a trend to varying overall survival (*p* = 0.06). Additionally, to the marker validation, we included methylation analyses for *BRCA1*. We could not identify any prognostic value for this parameter (Table 2) confirming data from the TCGA cohort and a recent meta-analysis [7, 53].

**Table 3:** Results of multivariate Cox analyses of clinical and molecular variables

Criteria	PFS			OS		
	HR	95% CI	*P*	HR	95% CI	*p*
FIGO I/II versus III/IV (*n* = 19 vs 158)	5.029	2.029–12.46	**0.001**	2.08	0.998–4.333	0.051
Resection R0 versus R1/2 (*n* = 41 vs 136)	1.865	1.257–2.766	**0.002**	2.453	1.591–3.781	**0.001**
*CAMK2N1* U versus M (*n* = 162 vs 15)	1.408	0.789–2.512	0.246	1.300	0.670–2.524	0.438
*KRT86* U versus M (*n* = 138 vs 39)	1.477	0.992–2.201	0.055	1.243	0.787–1.964	0.351
*CAMK2N1/KRT86* U versus M (*n* = 129 vs 48)	1.416	0.965–2.077	0.075	1.214	0.788–1.871	0.379
*CAMK2N1/RUNX3* U versus M (*n* = 147 vs 32)	1.587	1.031–2.442	**0.036**	1.602	1.017–2.542	**0.042**
*CAMK2N1/KRT86/RUNX3* U versus M (*n* = 119 vs 58)	1.543	1.070–2.223	**0.020**	1.348	0.903–2.012	0.144

Analyses included clinical variables and one of the molecular markers. Data for clinical variables are exemplarily and result from the analysis with *CAMK2N1/RUNX3*. LogRank test results with significance are marked in bold. U, unmethylated; M, methylated. Marker combinations are positive (methylated) if at least one single marker is methylated (<OR> combinations) otherwise the combination is scored as unmethylated

All methylation markers showing a significant prognostic value in univariate analysis (LogRank test) were separately analyzed together with both significant clinical parameters (FIGO and resection status) in multivariate Cox regression analysis (Table 3). We also analyzed clinical parameters with non-significant results in univariate analysis (histology, grading, treatment type) but they did neither exhibit prognostic value in multivariate analysis nor change results for the other clinical or molecular marker (data not shown). The combinations *CAMK2N1/RUNX3* and *CAMK2N1/RUNX3/KRT86* retained their significant prognostic value for PFS in this analysis. The calculated hazard ratio of progression for late stage EOC, after suboptimal debulking, for *CAMK2N1/RUNX3* methylation and for *CAMK2N1/RUNX3/KRT86* methylation was 5.029, 1.865, 1.587 and 1.543, respectively (Table 3). Thus, the above combinations of methylation markers had a prognostic value for PFS independent from FIGO staging and resection status. In relation to overall survival, only the resection status and *CAMK2N1/RUNX3* methylation showed an independent prognostic value (HR 2.45 and 1.60, respectively; Table 3). Late stage EOC exhibited a hazard ratio of 2.08 with *p* = 0.051.

### Explorative analysis of patients with defects in homologous recombination DNA repair (HRD)

A total of 10 patients with a familiar predisposition for EOC and a known mutation in *BRCA1*, *BRCA2* or *RAD51C* and available tumor tissue were included in the cohort. The information of the exact mutation was reported by genetic counseling (Additional file 1: Table S1). Additionally, we screened young EOC patients with available tissue for an exploratory somatic *BRCA1* mutation analysis and could identify five patients with mutations and included them in this cohort (*n* = 15, named HRD-defective EOC afterward, Additional file 1: Table S1).

**Fig. 2:**
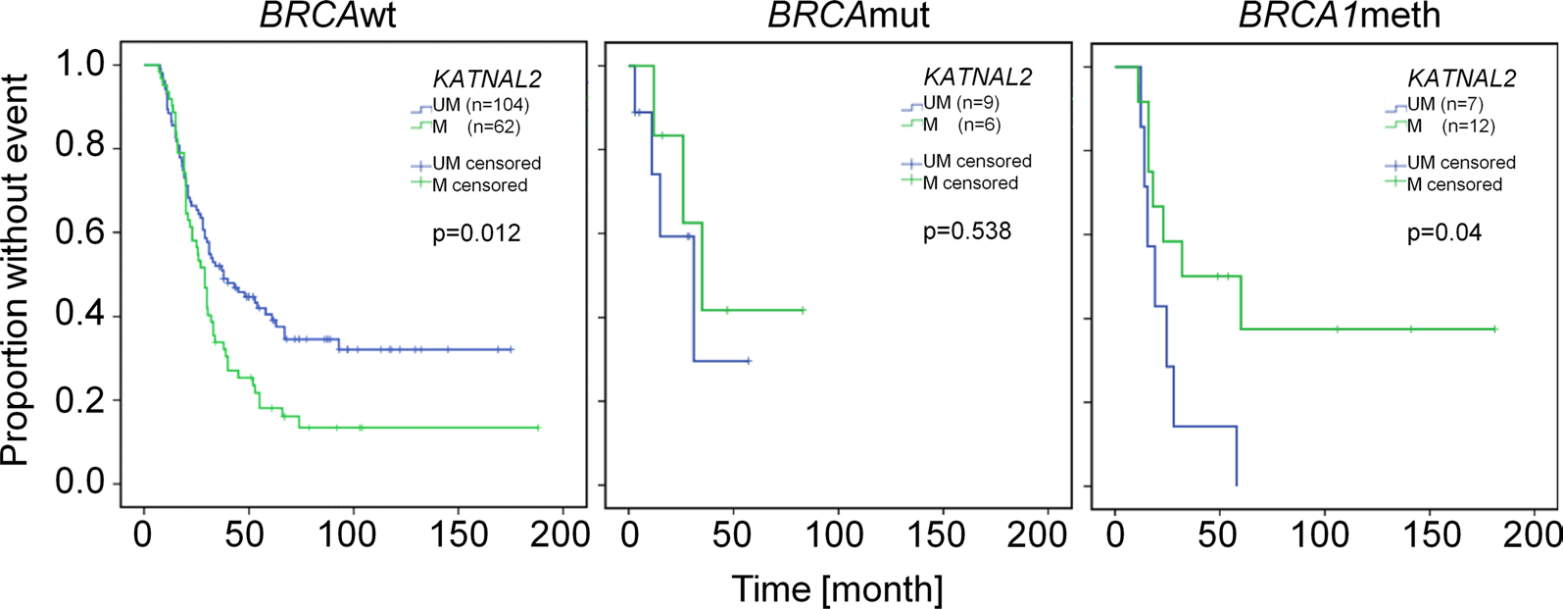
Kaplan–Meier plots of PFS for patients with sporadic EOC without *BRCA1* methylation (*BRCA1*wt), EOC with mutations in HRD genes (*BRCA*mut) or sporadic EOC with *BRCA1* methylation (*BRCA1*meth) stratified by *KATNAL2* methylation

Because of the small number of samples with mutations, all analyses have explorative character. We did not detect any significant differences in clinical variables between *BRCA*wt and HRD-defective EOC (data not shown). This is likely caused by the fact, that our cohort is enriched for high-grade serous EOC resembling *BRCA*-mutated EOC. Only *KRT86* showed significant different methylation frequencies in *BRCA*wt and HRD-defective EOC (22.9% and 0%, respectively; *p* = 0.044 Fisher’s exact Test). A trend of similar differences was seen for *CAMK2N1* and *BRCA1* (8.5% and 11.2% methylated vs. absent methylation in *BRCA*wt vs. HRD-defective EOC, respectively). However, no significant difference was detected in the combined methylation frequency for all genes (60% of samples in both groups). Additionally, the prognostic value for PFS did not strongly differ between *BRCA*wt and HRD-defective EOC for most methylation marker although the small sample size precluded significant results (data not shown). Interestingly, we observed a difference in prognosis of patients with *KATNAL2*-methylated *BRCA*wt and HRD-deficient tumors compared to the respective unmethylated group. Because HRD-deficiency can also be potentially caused by *BRCA1* methylation, we analyzed the prognostic value of *KATNAL2* methylation in *BRCA*wt EOC with and without *BRCA1* methylation and HRD-deficient EOC (Fig. 2). Whereas *KATNAL2* methylation showed no prognostic effect in the complete cohort (*n* = 188, Table 2) the stratification into *BRCA* aberration dependent groups resulted in significant prognostic effects (*p* < 0.05, LogRank test). *KATNAL2* methylation correlated with a significant worse outcome in patients with *BRCA*wt, but with a prolonged PFS in HRD-deficient and *BRCA1*-methylated EOC patients compared to the patients with respective *KATNAL2* unmethylated tumors (29 vs. 37 month (*p* = 0.012, HR 1.591), 35 vs. 31 month (n.s., HR 0.620) and 32 vs. 19 month (*p* = 0.04, HR 0.332), respectively; Fig. 2).

## Discussion

Aberrant DNA methylation can be used as molecular biomarker for disease diagnosis, prognosis or therapy stratification [58, 59]. Importantly, both the specific use and the target population must be defined and validated. Biomarker establishment and validation must balance between the power of the marker, the quality of the results and the homogeneity of the target population. In previous work, we identified 5 methylation markers that were prognostic for PFS by univariate analysis in a defined population—late stage type II EOC treated with platinum-taxane-based chemotherapy [44]. The aim of the present study was to analyze the potential independent prognostic value of these markers for PFS and OS in a group of EOC patients more heterogeneous in regard to tumor stage, grading, tumor histology and treatment by multivariate analyses. To provide excellent sample quality and correct sample classification, pathological evaluation of tissues was done. Because samples originated from two different medical centers, handling differences cannot be excluded, but the processing (cryo-sectioning, DNA purification), bisulfite conversion and MSP was done in one laboratory only. Moreover, the robustness of methylation analyses was determined by repeated assays from selected patients using different tissue blocks of the same tumor or repeated sections from identical biopsies resulting in an almost perfect agreement (Cohen’s kappa 0.881). We included samples with tumor cell fraction > 10% and did not observe a strong influence of the tumor content on methylation frequency of candidate regions (Additional file 1: Fig. S3). This may relate to the low cut-off value used to assign a positive methylation status (1% relative methylation, see method section) and absent methylation in normal keratinocytes, PBMCs and whole tissue sections of normal ovaries (data not shown). However, several limitations prohibit the transfer of the presented results to the general EOC population. First of all, the analyzed cohort does not represent all histological subtypes. Secondly, both median progression-free and overall survival are longer for our cohort compared to the general EOC population in Germany (31 month vs. 18.2 month PFS, 73 month vs. 44.1 month OS; [60]). The 5-year survival rate of 56% in our cohort is higher than the population-based rate in Germany (41%) [1]. Reasons for this are (1) the selection of platin-sensitive EOC and (2) the high proportion of optimal resected patients (macroscopically tumor-free, ~ 80%). A third limitation is that only a subset of patients had available information about germline mutation status (*n* = 10). An additional exploratory somatic *BRCA1* mutational analysis was done in early onset patients (age at diagnosis < 40 years) and revealed 5 patients with *BRCA1* mutations. Recent studies in Germany detected an approximately 20% *BRCA* germline mutation rate in unselected EOC patients (Harter [6]) suggesting that we underestimate the HRD-deficient fraction in our cohort (15/203, 7.4%). Nevertheless, this may only affect the prognostic value of *KATNAL2* (see below) because other markers seem to be not affected by HRD-status.

Using this enlarged, more heterogeneous cohort of EOC patients (Table 1) we could confirm the prognostic value of singular markers (*CAMK2N1*, *KRT86*) and combinations (*CAMK2N1/RUNX3*; *CAMK2N1/RUNX3/KRT86*) for PFS in univariate analysis. Additionally, both combinations are prognostic independent from tumor stage and resection status (multivariate Cox regression analysis). The stronger effect of combinations is not caused by the pure combination of markers potentially resulting in the detection of EOC with generally higher methylation level because EOC unmethylated for all analyzed marker showed no significant different survival compared to the group of EOC with at least one methylated marker (Table 2). It is more likely that informative methylation markers detect specific but different subgroups of EOC with reduced survival. This is supported by the low methylation frequency of informative markers (*CAMK2N1* 7.9%, *KRT86* 22.3%, *RUNX3* 14.9%). Whereas *CAMK2N1* and *RUNX3* methylation is independent from FIGO staging, *KRT86* methylation is not an independent prognostic factor but associated with late stage EOC. Thus, *KRT86* methylation can potentially be used for pre-operative staging if it can be specifically detected in systemic samples, e.g., blood. Besides the prognostic effect for PFS we could also detect an association of the marker combination *CAMK2N1/RUNX3* with overall survival both in univariate and multivariate analysis. EOC patients with methylation of *CAMK2N1* or *RUNX3* have a shorter overall survival than patients without methylation of these genes independently from FIGO staging or tumor resection status. The effect on overall survival is mainly caused by the shorter PFS in *CAMK2N1/RUNX3* methylation positive EOC patients (Fig. 1, Table 2) as Kaplan–Meier analyses for the time interval between relapse and death or last information available did not show a prognostic value for the marker combination (subset of relapsed patients *n* = 133 (70.7%); data not shown).

If prospectively validated *CAMK2N1/RUNX3* methylation may identify a subgroup of platin-sensitive patients that have worse prognosis. It can be speculated that this subgroup may benefit from maintenance treatment with Bevacizumab because both GOG-218 and ICON7 identified an overall survival benefit in a group of patients with high risk for progression differently defined by clinical variables [61, 62]. Although 29 patients of our cohort were treated with Bevacizumab, the number of methylation events (*n* = 2) is too low to draw any conclusion. However, published and unpublished data point to an influence of RUNX3 on the angiogenic potential of EOC by influencing the expression of thrombospondin-1 and other angiogenic factors ([63, 64], Heinze et al. in preparation). We also tried to identify an association of methylation marker status with *BRCA* status (*BRCA* mutation, *BRCA1* methylation) but did not detect a largely different prognostic value of *CAMK2N1/RUNX3* methylation in *BRCA*wt and potentially HRD-deficient EOC. Nevertheless, *KATNAL2* methylation being not prognostic in the complete group of EOC showed opposite effects in *BRCA*wt EOC without *BRCA1* methylation (HR 1.591, *p* = 0.012) and *BRCA*wt EOC with *BRCA1* methylation (HR 0.332, *p* = 0.04) or *BRCA*mut EOC (HR 0.620, n.s.). Although the numbers of patients with either *BRCA* aberration are small, this suggests different consequences of *KATNAL2* methylation in *BRCA*wt versus HRD-deficient EOC. Shared clinico-pathological features between *BRCA1*-mutated and *BRCA1*-methylated tumors may relate to the inactivation of BRCA1 albeit only BRCA1 mutations significantly influence patients survival [53]. Likely, we did not identify a prognostic value of BRCA1 methylation (Table 2). For patients with BRCA1-mutated tumors, survival analyses are limited by the low number and the shorter follow-up period for these patients (median mutated vs. wt: 36 month vs. 55 month). The PFS was not significantly different in *BRCA1*-mutated and *BRCA1*wt patients in our cohort (data not shown). In preliminary experiments, we analyzed BRCA1 protein expression by immunohistochemistry in a subset of samples and could observe significantly different staining patterns between *BRCA1*wt and mutated or methylated tumors (Additional file 1: Table S3) supporting earlier results from methylated tumors [52]. Both groups of tumors with BRCA1 aberration express lower levels of BRCA1. Thus, loss of BRCA1 may indeed result in the switch of the prognostic value of *KATNAL2* methylation. *KATNAL2* (Katanin Catalytic Subunit A1 Like 2) is a gene coding for a protein with microtubule binding and microtubule-severing ATPase activity whose detailed function in carcinogenesis is largely unknown. However, methylation of *KATNAL2* is a potential marker for severe cervical intraepithelial neoplasia [65]. The *Katanin* gene family regulates microtubule dynamics during mitosis, migration, ciliogenesis or cellular reorganization [66]. Strong *KATNAL2* downregulation leads to massive cell death, whereas mild downregulation causes microtubule stabilization and reduced turnover leading to mitotic defects and G2/M arrest in mouse cells [67]. Therefore, it can be speculated that microtubule stabilization by methylation-dependent *KATNAL2* downregulation induces mitotic defects, chromosome missegregation and chromosomal instability (CIN) potentially enabling tumor adaptation to chemotherapeutic intervention and worse outcome in *BRCA1*wt patients. However, BRCA proteins are not only involved in DNA repair but also microtubule organization—specifically in mitosis and cell polarization [68, 69]. Importantly, BRCA1 loss results in disturbed mitosis and CIN [70]. Thus, in tumors with *BRCA1* aberrations, the sum of mitotic defects may result in mitotic catastrophe, increased tumor cell death/better therapeutic response and improved survival. Besides the unknown basic mechanism for the different prognostic impact of *KATNAL2* methylation, depending on HRD status, this points to the potential necessity to differentiate between these EOC groups for biomarker identification and validation studies.

New biomarker candidates for cancer are identified frequently, a small subset of them will be published in peer-reviewed journals and only a few are validated in independent studies. However, even a positive validation does not guarantee a successful biomarker candidate [71]. Only ~ 0.8% of published methylation markers are commercially available [72]. Additional knowledge of the underlying mechanism of the biomarker’s detection and function would be helpful to underline its significance. One validation step for DNA methylation marker can consist of immunohistochemical detection of the epigenetically regulated protein. However, DNA methylation may not result in largely different expression a priori but rather restricts the capacity for an increased expression. Such regulation of the expression potential of affected genes was observed for genes hypermethylated during platin resistance development in EOC cell lines (i.e., *CAMK2N1* and *TRIB2*) in our laboratory [44, 73]. Both genes show gene expression differences between isogenic pairs of sensitive, unmethylated and resistant, hypermethylated cells under platin treatment only. Using the presently available chemotherapy-naïve EOC samples, we may not detect such changes in gene expression. Moreover, RUNX3 methylation affects only promoter P2 and the associated isoform that cannot be differentiated from other RUNX3 proteins by immunohistochemistry so far. Hence, we have not analyzed the protein expression of our candidate genes. However, first functional analyses of the biomarker candidate genes were conducted and a part of it is already published [56]. Specifically, the genes of the identified best methylation marker combination *CAMK2N1* and *RUNX3* were shown to have tumor suppressive functions (*CAMK2N1*) or to be involved in platin resistance and migration (*RUNX3*). CAMK2N1, one of two endogenous CAMKII inhibitors, was firstly identified as tumor suppressor inducing cell cycle arrest by p27 stabilization [74]. Accordingly, CAMK2N1 was not only described as tumor suppressor in EOC by our group but in multiple myeloma, oral squamous cell carcinoma, prostate cancer and thyroid cancer throughout the last years [75–78]. Therefore, a reduced PFS and OS of patients with *CAMK2N1*-methylated EOC vs. unmethylated EOC (*p* = 0.021 and *p* = 0.06, LogRank test) is in agreement with the gene function. *RUNX3* is described both as oncogene and tumor suppressor gene depending on the analyzed tumor entity or cellular background and readout [79, 80]. A tumor suppressive role of RUNX3 in vitro is described for Wilm’s tumor, prostate cancer, hepatocellular carcinoma, lung cancer and glioma [81–85]. In addition, we have shown dichotomous functions of the RUNX3 isoforms [56]. Importantly, methylation of *RUNX3* affects only promotor P2 and the associated transcript variant 2. This transcript variant increases cisplatin sensitivity and reduces cell migration for A2780 and cisplatin sensitivity of OVCAR3 in vitro ([56] and unpublished data). In the present study, we could not confirm a significant prognostic value of singular *RUNX3* methylation but a trend for decreased PFS for patients with tumors showing methylated *RUNX3* (median PFS 21 month vs. 31 month; *p* = 0.1 LogRank test).

## Conclusion

In conclusion, the retrospective analysis of 188 sporadic platinum-sensitive EOC proved an independent prognostic value of the methylation marker combination *CAMK2N1/RUNX3* for PFS and OS. If validated prospectively this combination may identify EOC patients with worse prognosis after standard therapy potentially benefiting from intensive follow-up, maintenance therapies or inclusion in therapeutic studies.

## Methods

### Patients material

A total of 203 fresh frozen (FF) and formalin-fixed paraffin-embedded (FFPE) tissue samples were used and divided into a validation and BRCA mutation cohort. The samples originated from the University Medical Center Hamburg-Eppendorf and the Jena University Hospital from 1996 to 2016. The use of patient’s material was approved by the Ethics committee Jena (#2582-6/09) and Hamburg (#190505). Clinical data were retrieved from the medical records. Progression-free survival was calculated from the date of primary surgery to first occurrence of relapse (confirmed by second-look surgery or non-invasive diagnostic tools) or last follow-up and overall survival was calculated from date of primary surgery until death or last follow-up. Surgical samples not needed for diagnostic procedures were snap-frozen in liquid nitrogen and stored at − 80 °C. The samples for the validation set were included based on the existence of three-years-follow-up data. Detailed and summary information on those two cohorts are depicted in Table 1 and Additional file 1: Table S1. Hematoxylin–Eosin stained sections of analyzed tissues were evaluated for histopathological classification and estimation of tumor cell fraction. Only tissues with > 10% tumor cells were used and 24.6%, 44.2% and 22.8% of samples contained 10–40%, 40–80% and > 80% tumor cells.

### DNA isolation

Sections from FF and FFPE tissue were used to perform analysis. The isolation was done according to the phenol-chloroform-isoamyl alcohol (PCI)-based DNA isolation protocol and using the QIAamp Kit according to the manufacture instruction.

### RNA isolation and cDNA synthesis for BRCA mutation detection

Sections from selected FF tissue were used for RNA isolation and subsequent cDNA synthesis. The isolation was done with the NucleoSpin® RNA kit according to the manual (Macherey–Nagel). 500 ng of RNA was reverse transcribed according to established techniques following published protocols [56].

### Bisulfite conversion

500 ng–1 µg of genomic DNA derived from tissue was converted using the EZ DNA Methylation-Gold™ according to the manual instruction (Zymo Research). The bsDNA concentration was determined and adjusted to 5 ng/µl. Identically treated gDNA from cervical swab of a healthy individual served as negative control sample. Positive control sample was created by in vitro methylation using the CpG-Methyltransferase (*M*.*Sss*I), according the supplier’s instruction (New England BioLabs). A serial dilution using in vitro methylated and unmethylated control bsDNA was created to generate the standard of 0%, 1%, 10% and 100% methylation level.

### Methylation-specific PCR

The q-MSP was performed according to [44] using the RotorGeneQ thermocycler (Qiagen, Germany) and candidate gene-specific primer listed in Additional file 1: Table S2 [44, 52]. All MSP assays detect DNA methylation within CpG islands located in the promoter/first exon region of associated genes except *KRT86*. The analyzed CpG island for *KRT86* is located at the 3’end (last exon) of the gene. Melting curve analyses confirmed the specificity of MSP amplification. The level of methylation was quantified stepwise by (1) calculating the relative amount (2^∆Ct^, relative methylation) of methylated target sequences in relation to a *beta-actin* fragment which was amplified by bisulfite-DNA-specific, but methylation-independent PCR (BS-PCR) and (2) comparing the relative methylation to an artificial dilution series (0–100% methylated DNA). Methylation data were evaluated qualitatively by application of a cutoff value and only samples showing a higher relative methylation as the 1% control were scored as methylated. Marker combinations reflect an <OR> combination of single marker results.

### BRCA1 mutation detection

To detect *BRCA1* exon mutations, a total of 6 PCRs were done using cDNA derived from patient’s RNA. Approximately 20 ng reverse transcribed nucleic acid were used. The reactions were conducted in 50 µl volume containing: dNTPs (240 µM each), forward and reverse primer (10 pmol each), DMSO (5%), MgCl_2_ (1.75 mM), AmpliTaqGold (1.25 U) and respective reaction buffer II (Applied Biosystems). The PCR steps were as follows: initial denaturation and activation at 95 °C for 10 min followed by 25 cycles of denaturation phase at 95 °C for 15 s, primer-specific annealing for 20 s at 56 °C and elongation at 72 °C for 30 s. The reaction products were size separated and sequenced in both directions. The readings were aligned to the *BRCA1* sequence according to human reference sequence (hg19).

### Statistical analysis

The statistical analysis was executed using SPSS and Microsoft Excel 2010/365. The unpaired student’s t-test was used for pairwise comparisons of continuous variables whereas chi-squared or Fishers Exact test was used for ordinal variables. The difference in survival between groups of patients was depicted by Kaplan–Meier plots and evaluated by LogRank test. Multivariate Cox regression analyses were used to calculated hazard ratios and determine the independent prognostic value of methylation markers.

## Supplementary information

**Additional file 1: Figure S1.** Graphical overview of methylations-specific PCR validation results. **Figure S2.** Kaplan–Meier plots for PFS and OS of EOC patients stratified by clinical variables. **Figure S3.** Comparison of methylation frequency in tumors with different tumor cell fraction. **Table S1.** Clinical data and mutation data of EOC with known mutations (*n* = 15). **Table S2.** Primer data for MSP and BRCA1 mutation analysis. **Table S3.** BRCA1 immunohistochemistry results for a subset of tumors depending on BRCA1 aberrations.

## Data Availability

All data generated or analyzed during this study are included in this published article [and its supplementary information files]. Raw data are available from the corresponding author on reasonable request.

## Abbreviations

Carbo: Carboplatin
DNMT: DNA-methyl-transferase
DNA: Deoxyribonucleic acid
EOC: Epithelial ovarian cancer
FIGO: International Federation of Gynecology and Obstetrics
HBOC: Hereditary breast and ovarian cancer syndrome
HGSOC: High-grade serous ovarian cancer
HRD: Homologous DNA repair defects
M: Methylated
MBD: Methyl-binding domain protein
MSP: Methylation-specific PCR
OS: Overall survival
Pac: Paclitaxel
PARP: Poly-ADP-ribose polymerase
PARPi: PARP inhibitor
PFS: Progression-free survival
U: Unmethylated
