# Supplementary material for: CAMK2N1/RUNX3 methylation is an independent prognostic biomarker for progression-free and overall survival of platinum-sensitive epithelial ovarian cancer patients
Source: Clin Epigenetics. 2021 Jan 22;13:15. doi: 10.1186/s13148-021-01006-8 (PMC7824928; doi:10.1186/s13148-021-01006-8)
Supplement: Supplementary file 1 — Additional file 1: Figure S1. Graphical overview of methylations-specific PCR validation results. Figure S2. Kaplan–Meier plots for PFS and OS of EOC patients stratified by clinical variables. Figure S3. Comparison of methylation frequency in tumors with different tumor cell fraction. Table S1. Clinical data and mutation data of EOC with known mutations (n = 15). Table S2. Primer data for MSP and BRCA1 mutation analysis. Table S3. BRCA1 immunohistochemistry results for a subset of tumors depending on BRCA1 aberrations. [file 13148_2021_1006_MOESM1_ESM.pdf]

Suppl. Figure S1

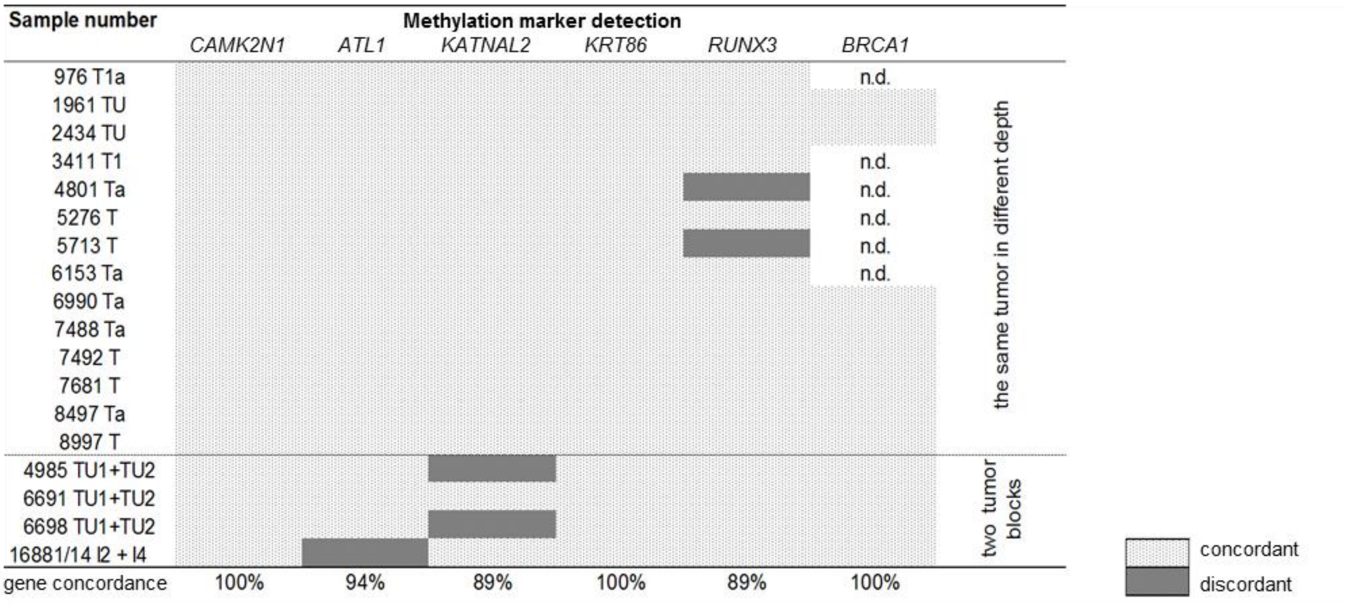

Figure S1: Validation of MSP assays. Different sections of the same tissue block (upper part) or different tumor blocks of the same patient (lower part) were analyzed for marker methylation by MSP. Concordant and discordant analyses are marked in grey and dar-grey, respectively. The gene-specific concordance ranges from 89%-100% and averages to 95% overall concordance (Cohen´s Kappa = 0.881).

Suppl. Figure S2

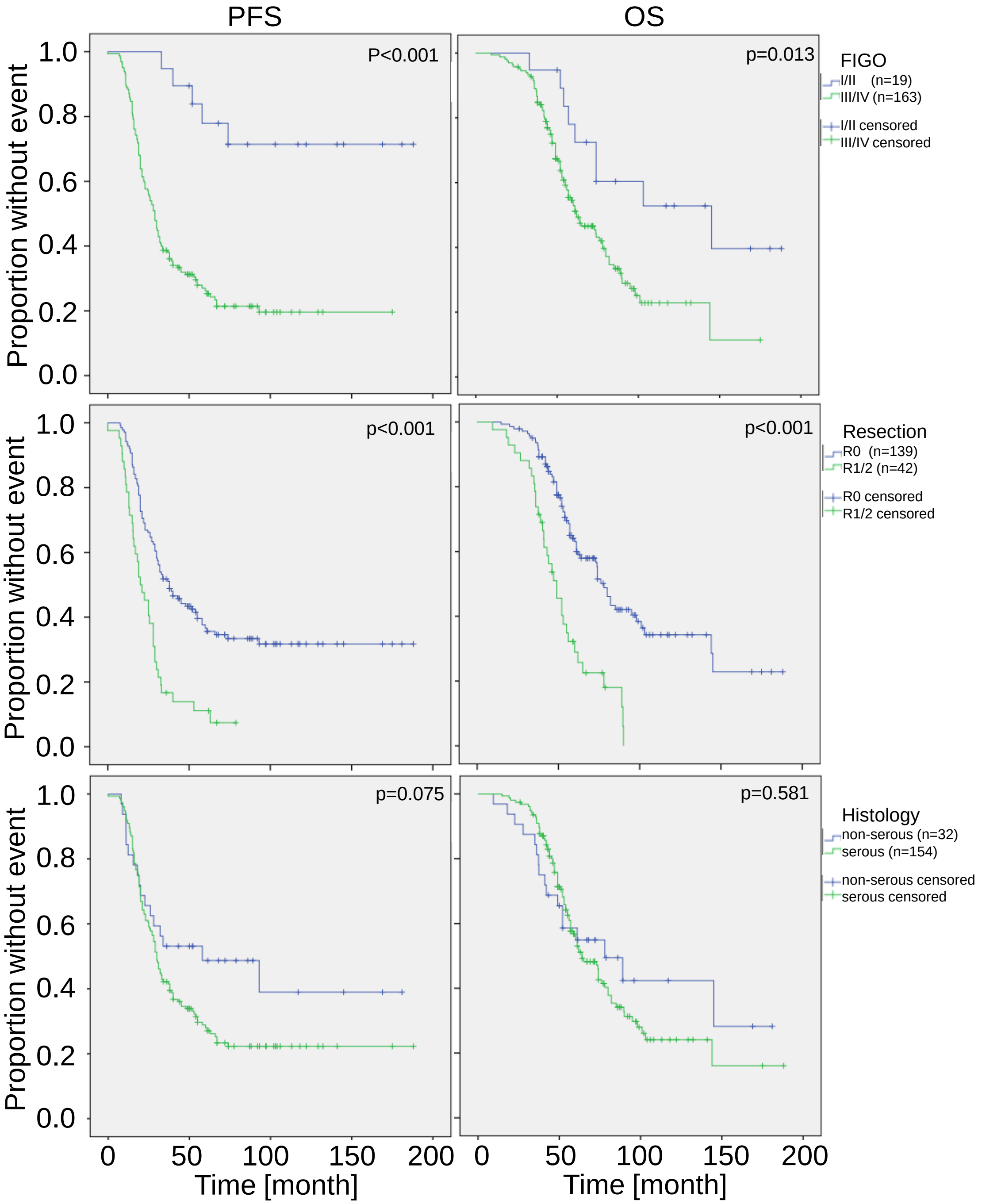

Figure S2: Kaplan-Meier plots for PFS und OS for 188 EOC patients stratfied by FIGO stage, resection status and histology. LogRank test was utilized for statistical evaluation.

Suppl. Figure S3

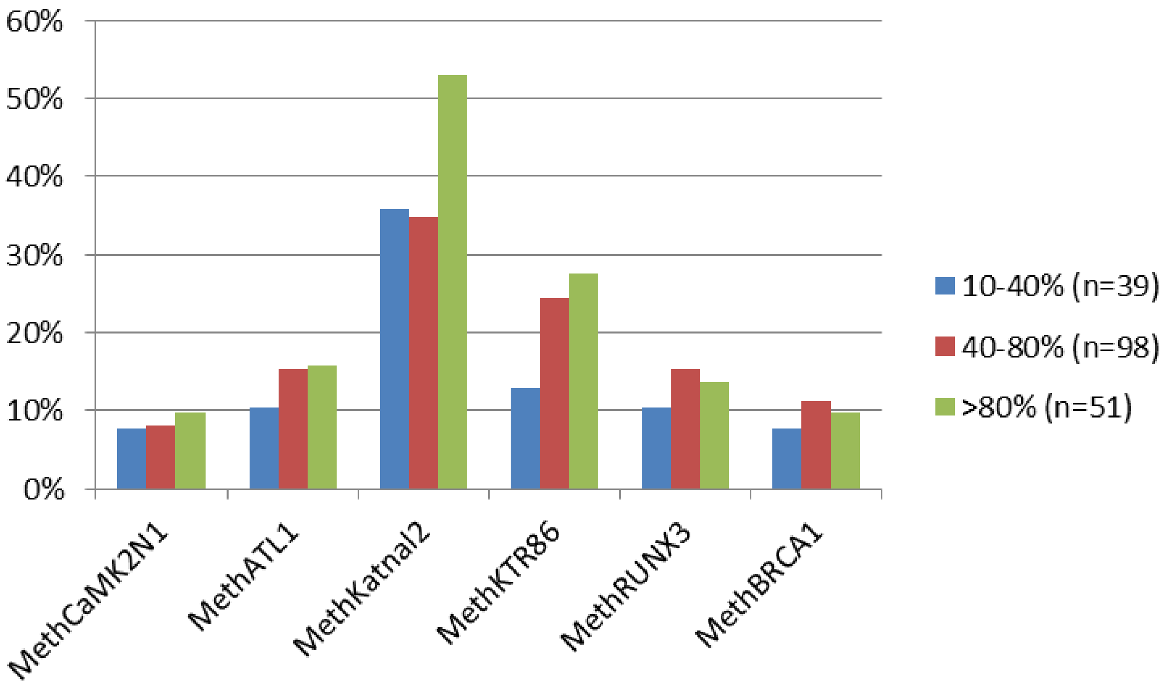

Figure S3: Methylation frequency of candidate genes depending on tumor cell fraction of tissues.

Suppl. Table S1. Data from EOC patients with HRD mutations

sample	age	histo	pT	pN	pM	FIGO	G	R	mutation
3325	39	serous	1c	0	x	IC	1	0	BRCA1 c.1319T>C[S#]
8265	48	mucinous	3c	1	1	IV	3	0	BRCA1 truncated[S]
8644	27	mucinous	1a	1	0	IA	1	0	BRCA1 truncated[S]
8861	49	serous	3c	x	0	IIIC	3	2	BRCA1 c.5503C>T [H]
9473	48	serous	3c	0	1	IIIC	3	0	BRCA1 c.3627 ins A[S]
9877	36	serous	3b	1	0	IIIC	2	0	BRCA1 c.1067A>G [S#]
10003	63	serous	3b	1	0	IIIC	3	0	BRCA1 mut
10080	73	serous	3c	x	0	IVA	3	2	BRCA2 c.5238dupT [H]
10163	53	mucinous	3c	1	1	IIIC	3	0	BRCA2 mut [H]
10196	44	serous	3c	x	1	IIIC	3	2	BRCA2 c.1953_1956del[H]
16881/14	60	serous	3c	0	0	IIIC	3	0	BRCA1 c.1953_1956del[H]
17571/15	49	serous	3c	1	0	IIIC	3	0	BRCA1 c.5062G>T [S]
2878/16	45	serous	3c	1	0	IIIC	2	0	BRCA1 c.5266depC [H]
14009/17	46	serous	1c	0	0	IC	1	0	RAD51C [H], CHEK2 [H]
14426/17	49	serous	3c	1	0	IIIC	3	0	BRCA1 c.5080G>A [H]

H heterozygote, # unknown pathogenicity, S somatic mutation determined via exon analysis

Table S2 Overview of applied primer for MSP and *BRCA1* mutation analysis.

Primer	Oligo sequence	product size	melting temp.	Localization*
Actin beta-BS-F<br>Actin-beta BS-R	TGGTGATGGAGGAGGTTTAGTAAGT<br>AACCAATAAAACCTACTCCTCCCTTAA	125bp	60 °C	no CpG island, 1500nt upstream 1st exon
ATL1-M-F<br>ATL1-M-R	AATTGCGTTTAGCGCGGGTAC<br>CGATAAAACGCTATCACTACGCTCG	89bp	61 °C	CpG island (CpG:52); promoter+1st exon
BRCA1-M-F<br>BRCA1-M-R	GCGAGTTTACGTCGCGTAGTC<br>AATTTCCGTAACAACGAAAAAAACG	84bp	60 °C	CpG island; promoter+1st exon
Katnal2-M-F<br>Katnal2-M-R	TTATTGTTGTTGCGGGACGATTTC<br>AAATTTAACCACCTCGACCGAACG	109bp	61 °C	CpG island (CpG:23); 1st exon
KRT86-M-F<br>KRT86-M-R	TTTAGTTACGGGGGTTCGGC<br>GCCACCGCGAAAACTACTAACG	256bp	62 °C	CpG island (CpG:63); last exon
CAMK2N1-M-F<br>CAMK2N1-M-R	TAGTCGTTGGTCGATTGATTC<br>AACCTCGATCGACTACCG	184bp	60 °C	CpG island (CpG:245); promoter+1st exon
RUNX3-M-F<br>RUNX3-M-R	GTGTTTCGATTTCGGTTTAGCGTC<br>CGCTCGACTCCGAAACCTCG	353bp	64 °C	CpG island (CpG:311); promoter+1st exon
RUNX3-Pa-M-F<br>RUNX3-Pa-M-R	TTAGCGTCGTTCGGTAAAATTTC<br>ACCGCGAATAAAATACGAACG	127bp	60 °C	CpG island (CpG:311); promoter+1st exon
BRCA1-ORF1-F<br>BRCA1-ORF1-R	CACCCTCTGCTCTGGGTAAA<br>GCTGTAATGAGCTGGCATGA	893bp	56 °C	
BRCA1-ORF2-F<br>BRCA1-ORF2-R	AGCTGAGAGGCATCCAGAAA<br>TGCTCCGTTTGGTTAGTTCC	864bp	56 °C	
BRCA1-ORF3-F<br>BRCA1-ORF3-R	AAAGCAGATTTGGCAGTTCAA<br>GTCCCTTGGGGTTTTCAAAT	883bp	56 °C	
BRCA1-ORF4-F<br>BRCA1-ORF4-R	GGAAGGCAAAAACAGAACCA<br>CTCAGGTTGCAAAACCCCTA	902bp	56 °C	
BRCA1-ORF5-F<br>BRCA1-ORF5-R	CAGAGGGCCAAAATTGAATG<br>TCACTCTCACACCCAGATGC	900bp	56 °C	
BRCA1-ORF6-F<br>BRCA1-ORF6-R	CAAGAAGAGCAAAGCATGGA<br>CAGACACCACCATGGACATT	901bp	56 °C	
BRCA1-ORF7-F<br>BRCA1-ORF7-R	TGCTGGGTATAATGCAATGG<br>AAGCTCATTCTTGGGGTCCT	775bp	56 °C	

* UCSC CpG island names parenthesized if available

Suppl. Tab. S3 BRCA1 immunhistochemistry results depending on BRCA1 aberrations*

			BRCA1 aberration			total
			wt	mut	meth	
BRCA1_IHC	negativ	n	2	3	2	7
		% of samples	8%	23%	67%	17%
	weak positive	n	6	6	1	13
		% of samples	24%	46%	33%	32%
	positive	n	17	4	0	21
		% of samples	68%	31%	0%	51%
total		n	25	13	3	41
		% of samples	100%	100%	100%	100%

* Chi^2 test $p<0.05$
